# *Et*G6PI Is Implicated in Host Cell Invasion and Maduramycin Resistance in *Eimeria tenella*

**DOI:** 10.3390/microorganisms14061204

**Published:** 2026-05-26

**Authors:** Fanghe Zhao, Yu Yu, Ke Xiao, Qiping Zhao, Shunhai Zhu, Jinwen Wang, Jiayu Bai, Wenqi Han, Shanbo Wu, Hui Dong, Hongyu Han

**Affiliations:** Key Laboratory of Animal Parasitology of Ministry of Agriculture, Shanghai Veterinary Research Institute, Chinese Academy of Agricultural Sciences, Shanghai 200241, China; xzfh824@163.com (F.Z.); yuyushvri@163.com (Y.Y.); xiaokecaas@163.com (K.X.); zqp@shvri.ac.cn (Q.Z.); zhushunhai@shvri.ac.cn (S.Z.); 82101221311@caas.cn (J.W.); baijiayu0163@163.com (J.B.); hwq0714@163.com (W.H.); wushanbo@163.com (S.W.)

**Keywords:** coccidiosis, *Eimeria tenella*, drug resistance, glucose-6-phosphate isomerase

## Abstract

Chicken coccidiosis, caused by obligate intracellular protozoa of the genus *Eimeria*, inflicts substantial economic losses on the global poultry industry. The extensive use of anticoccidial drugs has led to widespread resistance, underscoring the need for molecular markers associated with this resistance. Our previous RNA-seq analysis revealed differential expressions of glucose-6-phosphate isomerase (*Et*G6PI) between drug-sensitive (DS) and maduramycin-resistant (MRR) strains of *Eimeria tenella*. In this study, we examined *Et*G6PI expression across developmental stages using qPCR and Western blotting, finding that both transcription and translation peaked in second-generation merozoites. Furthermore, *Et*G6PI expression was significantly upregulated in MRR strains in a dose-dependent manner and was also elevated in field isolates exhibiting maduramycin resistance. Indirect immunofluorescence localized *Et*G6PI to the parasite surface, cytoplasm, and parasitophorous vacuole membrane (PVM), with signal intensity increasing during intracellular development. In addition, anti-r*Et*G6PI polyclonal antibodies significantly inhibited sporozoite invasion of host cells in vitro. These results indicate that *Et*G6PI plays a role in host cell invasion, a process essential for parasite proliferation, and is associated with maduramycin resistance in *E. tenella*, supporting its potential as a biomarker for resistance detection in field settings.

## 1. Introduction

Chicken coccidiosis is a common intracellular parasitic disease caused by *Eimeria* spp. infection of the intestinal tract. It severely damages the poultry intestine, leading to symptoms such as diarrhea, blood in the feces and even death [1]. Currently, ten species of *Eimeria* are recognized as infectious in chickens, each exhibiting different pathogenicity depending on the infection site. Among them, *Eimeria tenella* is the most pathogenic and harmful. However, mixed infections with several *Eimeria* species are frequently observed in daily production [2]. Current control of coccidiosis relies mainly on anticoccidial drugs and live oocyst vaccines [3]. While live vaccines are widely used in laying hens and breeders, their application in broilers is limited. This is because broilers have a short rearing cycle, and furthermore, vaccination requires time to establish immunity while potentially causing short-term side effects that impair performance. Consequently, anticoccidial drugs remain the primary control method in broiler production [3]. Due to the long-term and irrational use of drugs, coccidia have developed resistance to almost all drugs used [4]. Nevertheless, the mechanisms of coccidial drug resistance development are currently not well understood.

Drug resistance mechanisms were investigated by analyzing gene transcription and protein translation. Studies in other apicomplexan parasites have shown that resistant strains differentially express certain proteins compared to sensitive strains [5]. This differential expression may be driven by changes in regulatory genes, as suggested by subsequent research [6]. Similarly, in other protozoa, such as *Plasmodium*, differential gene expression has been linked to drug resistance development [7]. To investigate whether a similar mechanism exists in *E. tenella*, we conducted transcriptome sequencing on a sensitive strain (DS) and maduramycin-resistant (MRR) strain which was induced from the DS strain under laboratory conditions using progressive drug pressure [8,9]. Our analysis revealed that *E. tenella* glucose-6-phosphate isomerase (*Et*G6PI; ToxoDB Accession number, ETH_00018515) was significantly upregulated in MRR sporozoites compared to the DS. This finding suggests that its upregulation may be associated with resistance to maduramycin.

Glucose-6-phosphate isomerase (G6PI) is a multifunctional cytosolic enzyme. Its primary role is to catalyze the interconversion between D-glucose-6-phosphate and D-fructose-6-phosphate, playing a crucial part in glycolysis and gluconeogenesis [10]. Beyond its canonical enzymatic role in glycolysis, G6PI also exhibits non-glycolytic functions, such as acting as a growth factor and being implicated in processes like the invasive growth of rheumatoid arthritis synovial tissues [11,12]. However, its specific function in *E. tenella* has not been elucidated. Notably, in cancer biology, enhanced glycolysis is closely associated with the induction of drug resistance [13]. Since glycolysis serves as the primary energy source during the endogenous development of *E. tenella*, we hypothesize that the differential expression of *Et*G6PI in the maduramycin-resistant strain may be associated with the emergence of drug resistance.

Despite the identification of several differentially expressed genes, the functional roles of most candidate resistance-associated proteins, including *Et*G6PI, remain unexplored. This study aims to fill this gap by characterizing *Et*G6PI expression, localization, and its potential involvement in host cell invasion and drug resistance. This study conducted a preliminary investigation of *Et*G6PI, a glycolysis-related gene upregulated expression in maduramycin-resistant *E. tenella*. We first characterized its expression profile across developmental stages of the DS strain using qPCR and Western blotting and determined its subcellular localization via immunofluorescence analysis. An in vitro inhibition assay was employed to assess its functional role in host cell invasion. Additionally, we examined *Et*G6PI expression under varying drug pressures and in field isolates. Collectively, these findings implicate *Et*G6PI not only in core glycolysis but also in host cell invasion and the drug resistance phenotype, thereby providing a basis for further research into its functional mechanisms.

## 2. Materials and Methods

### 2.1. Ethics Statements

Animals are used in accordance with a protocol approved by the Animal Care and Use Committee of the Shanghai Veterinary Research Institute of the Chinese Academy of Agricultural Sciences.

### 2.2. Animals and Parasites

One-day-old chickens were obtained from a local farm (Fengxian, Shanghai, China) and housed at the Shanghai Veterinary Research Institute. The chickens were provided with a standard diet and water without coccidia. Additionally, New Zealand rabbits were purchased from Shanghai Jiagan Biotechnology Limited Company (Shanghai, China).

The *E. tenella* Shanghai DS strain (Resource No. CAAS2111160721), originally isolated from a Shanghai poultry farm in the 1980s and maintained in our laboratory, served as the base strain. Using the DS strain as the progenitor, we generated a MRR strain (Resource No. CAAS21111609) which is resistant to 7.0 ppm maduramycin through progressive drug pressure in the laboratory [8]. Additionally, we maintained graded resistance strains for maduramycin (3.0 and 5.0 ppm). All strains are routinely passaged every six months to preserve viability. Different developmental stages of *E. tenella* were obtained as follows. Unsporulated oocysts and sporulated oocysts were collected and purified as previously described [14]. Sporozoites were purified from sporulated oocysts by in vitro excystation [15]. Second-generation merozoites were isolated from the chicken cecal mucosa at 112 h post-inoculation and further purified via Percoll gradient centrifugation [16].

The DF-1 chicken embryo fibroblast cell line (ATCC CRL-12203) was cultured in Dulbecco’s Modified Eagle’s Medium (DMEM; Gibco™, Thermo Fisher Scientific, Waltham, MA, USA) for immunofluorescence assays and in vitro inhibition assay.

### 2.3. Cloning and Bioinformatics Analysis of Gene EtG6PI

Total RNA was extracted from the sporulated oocysts of *E. tenella* DS strain using TRIzol reagent (Invitrogen™, Thermo Fisher Scientific, Waltham, MA, USA) according to the manufacturer’s instructions. Following quantification and integrity verification, the total RNA was reverse-transcribed into cDNA using the RevertAid First Strand cDNA Synthesis Kit (Vazyme, Nanjing, China). The open reading frame (ORF) of *Et*G6PI was amplified using this cDNA as the template. The amplification product was purified and ligated into the pGEM-T Easy vector (Promega Corporation, Madison, WI, USA) for sequencing.

The sequence of *Et*G6PI was analyzed using the BLAST program on the National Center for Biotechnology Information (http://www.ncbi.nlm.nih.gov/BLAST/ (accessed on 6 January 2025)). The molecular mass was found using ToxoDB (https://toxodb.org/toxo/app/record/gene/ETH_00018515 (accessed on 6 January 2025)). The theoretical isoelectric point (pI) and molecular weight (Mw) were calculated with a Compute pI/Mw tool (https://web.expasy.org/compute_pi/ (accessed on 6 January 2025)). The transmembrane region was predicted by TMHMM (http://services.healthtech.dtu.dk/services/TMHMM-2.0/ (accessed on 6 January 2025)). Functional structural domains of amino acid sequences were analyzed through scanprosite (https://prosite.expasy.org (accessed on 6 January 2025)). Signal peptide prediction was performed using SignalP5.0 (www.cbs.dtu.dk/services/SignalP (accessed on 6 January 2025)).

A three-dimensional homology model of *Et*G6PI was constructed using the SWISS-MODEL server (https://swissmodel.expasy.org/ (accessed on 13 May 2026)), based on the crystal structure of substrate-bound G6PI from *Toxoplasma gondii* (PDB ID: 3UJH; sequence identity: 59.48%). Model quality was assessed using the Global Model Quality Estimation (GMQE) and QMEANDisCo scores.

Homologous protein sequences were aligned using the ClustalW program integrated in MEGA12 software. All the amino acid sequences analyzed were derived from apicomplexan parasites. Based on the G6PI protein sequences, a phylogenetic tree was constructed by the Maximum Likelihood Tree method, and the visualization and subsequent processing of the tree were completed using MEGA12 software.

### 2.4. Expression of Recombinant EtG6PI and Preparation of Polyclonal Antibodies

The *Et*G6PI ORF was cloned into the pGEX-4T-2 vector (Novagen, Darmstadt, Germany) and transformed into *E. coli* BL21(DE3) for recombinant protein expression induced by 1.0 mM IPTG. After induction, bacterial cells were collected by centrifugation, lysed by ultrasonication, and analyzed by SDS-PAGE to evaluate expression levels and approximate molecular weight. The recombinant protein (r*Et*G6PI) was purified via nickel-ion affinity chromatography. Protein purity and concentration were validated by SDS-PAGE and BCA assay (Beyotime, Shanghai, China), respectively, and the purified aliquots were stored at −20 °C.

This purified r*Et*G6PI was used as an antigen to immunize New Zealand rabbits at a dose of 200 µg per rabbit per immunization. The immunization regimen began with a primary subcutaneous injection of r*Et*G6PI emulsified in Freund’s complete adjuvant (Sigma-Aldrich, Darmstadt, Germany), followed by a booster on day 14 and three additional weekly boosters, all using Freund’s incomplete adjuvant (Sigma-Aldrich, Darmstadt, Germany). Serum was collected one week after the final immunization, and IgG antibodies were subsequently purified from the antiserum using Protein A + G Agarose (Beyotime, Shanghai, China).

### 2.5. Quantification of EtG6PI mRNA Transcription Levels by qPCR

*Et*G6PI transcription was quantified by qPCR, initially across four developmental stages (unsporulated oocysts, sporulated oocysts, sporozoites, and second-generation merozoites) of the DS strain. Additionally, we compared *Et*G6PI transcription in sporozoites exposed to varying concentrations of maduramycin between the DS and MRR strains. Gene-specific primers for *Et*G6PI (FP: 5′-AGT CGC CAC TGA GGG TTT CAT TTG-3′; RP: 5′-GAT GTC CTT CGC CAG CAC CTT C-3′) were designed using Primer 6.0, with 18S rRNA serving as the internal reference.

Total RNAs were extracted from all samples using TRIzol reagent (Invitrogen™, Thermo Fisher Scientific, Waltham, MA, USA) and further purified with the RNeasy Mini Kit (Qiagen, Hilden, Germany). First-strand cDNAs were then synthesized using the M-MLV Reverse Transcription Kit (Invitrogen). qPCR was performed on a QuantStudio 5 system (Thermo Fisher, Waltham, MA, USA) using the SYBR Premix DimerEraser kit (TaKaRa, Kyoto, Japan). Each sample was analyzed in triplicate, and the entire experiment was independently repeated three times. Relative gene expression was calculated using the 2^−ΔΔCt^ method.

### 2.6. Western Blot Analysis

Proteins were extracted from unsporulated oocysts, sporulated oocysts, sporozoites, and second-generation merozoites of the *E. tenella* DS strain using RIPA buffer (Beyotime, Shanghai, China) with protease inhibitors (Sigma-Aldrich, Darmstadt, Germany). Protein concentrations were determined with a BCA kit (Beyotime, Shanghai, China), and equal amounts of total protein were separated by SDS-PAGE and transferred onto PVDF membranes. After blocking with 5% skimmed milk, membranes were incubated simultaneously with rabbit anti-r*Et*G6PI polyclonal antibody (1:200) and mouse anti-α-tubulin monoclonal antibody (1:5000, loading control) at 37 °C for 2 h, followed by incubation with corresponding HRP-conjugated secondary antibodies (1:5000) at room temperature for 1 h. All protein signals were detected using the SuperSignal™ West Pico Plus Chemiluminescent Substrate (Thermo Fisher, Waltham, MA, USA) and visualized with a ChemiDoc™ Touch Imaging System. The relative intensities of the target protein bands were quantified via grayscale analysis using ImageJ 1.54g software.

The immunoreactivity of r*Et*G6PI was also analyzed. The blot was probed with the following primary antibodies: rabbit anti-sporozoite serum and serum from healthy rabbits (as a negative control).

### 2.7. Indirect Immunofluorescence Localization of EtG6PI

DF-1 cells were seeded onto glass coverslips placed in a six-well plate and cultured overnight. Purified sporozoites of the DS strain were then added at a parasite-to-cell ratio of 3:1 and incubated at 41 °C with 5% CO_2_ to permit invasion. Coverslips were collected at specified time points post-infection (2, 12, 24, 60, and 72 h). Separately, purified sporozoites and second-generation merozoites were individually smeared onto coverslips and air-dried for extracellular analysis. All coverslip samples (infected cells and parasite smears) were fixed with 4% paraformaldehyde for 15 min and permeabilized with 0.1% Triton X-100 for 10 min. After PBS washes, non-specific sites were blocked with 2% BSA at room temperature for 1 h. Samples were incubated with rabbit anti-r*Et*G6PI polyclonal antibody (1:200 in 2% BSA) at 37 °C for 2 h, followed by thorough washing and incubation with FITC-conjugated goat anti-rabbit IgG (1:500) at 37 °C for 1 h in the dark. Nuclei were counterstained with DAPI (10 µg/mL) at room temperature for 15 min. After a final wash, coverslips were mounted with an anti-fade medium. Fluorescence images were acquired using a fluorescence microscope to determine the subcellular localization of *Et*G6PI in *E. tenella*.

### 2.8. In Vitro Inhibition Assay

To assess the inhibitory effect of anti-r*Et*G6PI IgG on host cell invasion, an in vitro assay was performed. DF-1 cells were first seeded in a 24-well plate and cultured overnight. Following the standard protocol for antibody neutralization assays of *E. tenella* sporozoite invasion [17,18], fresh sporozoites of *E. tenella* DS strain were labeled with CFDA SE and then pre-incubated with purified rabbit anti-r*Et*G6PI IgG at different concentrations (50–400 µg/mL) for 2 h, using healthy rabbit IgG and untreated parasites as negative and untreated controls, respectively. After pre-incubation, 6 × 10^5^ sporozoites per well were added to the cell monolayers (in triplicate) and incubated for 8 h to facilitate invasion. The cells were then harvested and analyzed by flow cytometry. Invasion inhibition rates of each treatment group were calculated relative to the untreated control group. Due to the polyclonal nature of the antibody, the specificity of the inhibitory effect is inferred from the dose-dependent response and the absence of inhibition by control IgG.

### 2.9. Detection Transcription Level of EtG6PI in Field Samples

To explore the feasibility of *Et*G6PI as a potential molecular marker for detecting resistance to maduramycin and other anticoccidial drugs, this study was conducted on three field isolates, Nantong F15 (NF15), Nantong A (NA), and Anhui 1 (A1). These isolates were obtained by passaging oocysts from fecal samples collected from intensive poultry farms in Jiangsu and Anhui Provinces, China. Drug susceptibility was assessed using four standard metrics (anticoccidial index, ACI; reduction in lesion score, RLS; relative oocyst production, ROP; percentage of optimum anticoccidial activity, POAA). The three isolates exhibited complete resistance to diclazuril, maduramycin and salinomycin [19]. To analyze *Et*G6PI expression, unsporulated oocysts were harvested from cecal contents of both drug-treated and untreated chickens infected with these isolates. Following sporulation and sporozoite purification, *Et*G6PI mRNA transcriptal levels were quantified by qPCR.

### 2.10. Statistical Analysis

All data are presented as the mean ± SD. The results of qPCR and in vitro inhibition assay were calculated and then *t*-test and one-way ANOVA were performed for comparison between groups using the software package GraphPad Prism 6, with statistical graphs plotted accordingly. Student’s *t*-test was used for two-group comparisons; one-way ANOVA followed by Tukey’s post hoc test was used for multi-group comparisons. *p*-values < 0.05 were considered statistically significant.

## 3. Results

### 3.1. Cloning and Analysis of the EtG6PI Coding Sequence

**Figure 1:**
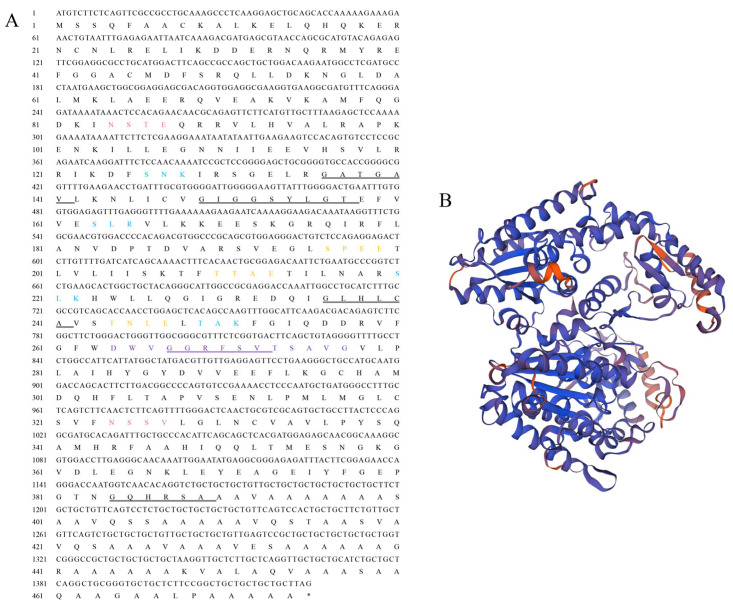
Bioinformatic analysis of *Et*G6PI. (**A**), amino acid sequence analysis. Purple, glucose-6-phosphate isomerase (GPI) family signatures and profile; pink, N-glycosylation site; blue, protein kinase C phosphorylation site; double horizontal, N-myristoylation site; orange, casein kinase II phosphorylation site. (**B**), a three-dimensional homology model of *Et*G6PI.

Sequence analysis of *Et*G6PI revealed that the ORF is 1422 bp in length, encoding 473 amino acid residues with a predicted molecular mass of 50.3 kDa and a theoretical isoelectric point of 6.84. SignalP and TMHMM predicted no signal peptide or transmembrane domain. The results of protein structure and function domain prediction showed that *Et*G6PI possesses one glucose-6-phosphate isomerase (GPI) family signatures and profile (264–277), two N-glycosylation site (84–87, 324–327), four protein kinase C phosphorylation site (126–128, 163–165, 220–222, 249–251), six N-myristoylation site (136–141, 149–154, 152–157, 236–241, 267–272, 384–389) and three Casein kinase II phosphorylation site (196–199, 210–213, 244–247) (Figure 1A). Homology modeling revealed that *Et*G6PI adopts a canonical G6PI fold with conserved α/β and α-helical domains and is predicted to form a homo-dimer (Figure 1B). The model showed good quality (GMQE 0.70, QMEANDisCo 0.79), supporting its conserved catalytic function in glycolysis.

BLASTp analysis revealed that the protein exhibits 100% identity with GPI of *E. tenella* (GenBank accession no. XP_013233195.1) and shares 95.76%, 73.01%, 72.52%, 71.72%, and 70.22% identity with glucose-6-phosphate isomerase proteins from *E. necatrix* (GenBank accession no. XP_013440080.1), *E. acervuline* (GenBank accession no. XP_013249516.1), *E. magna* (GenBank accession no. KAL8444139.1), *E. maxima* (GenBank accession no. XP_013333039.1), and *E. stiedai* (GenBank accession no. KAL8275620.1), respectively.

**Figure 2:**
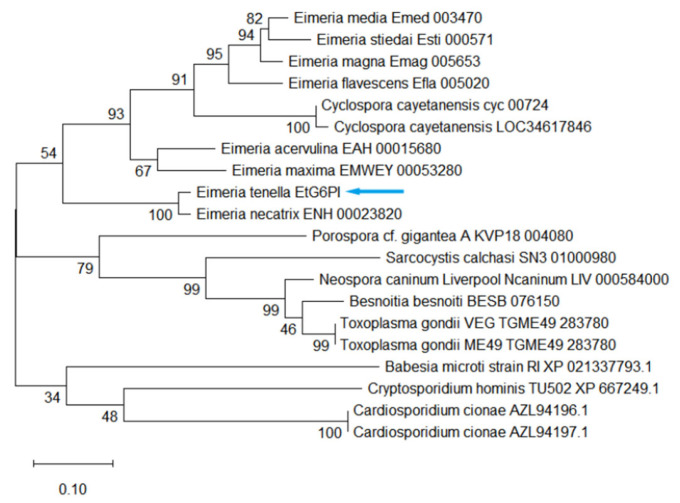
Phylogenetic tree of G6PI protein from different species. The *Et*G6PI sequence from *E. tenella* is highlighted with a blue arrow.

Phylogenetic analysis of *Et*G6PI revealed that G6PI sequences of *Eimeria* spp. formed a highly conserved core clade among apicomplexan parasites with intra-generic bootstrap values of 82–100 indicating extreme sequence conservation and a close phylogenetic relationship with *Cyclospora cayetanensis* and showed a distinct evolutionary divergence from other apicomplexan parasites such as *Toxoplasma*, *Sarcocystis*, and *Babesia* (Figure 2).

### 3.2. Expression and Reactogenicity Analysis of rEtG6PI

**Figure 3:**
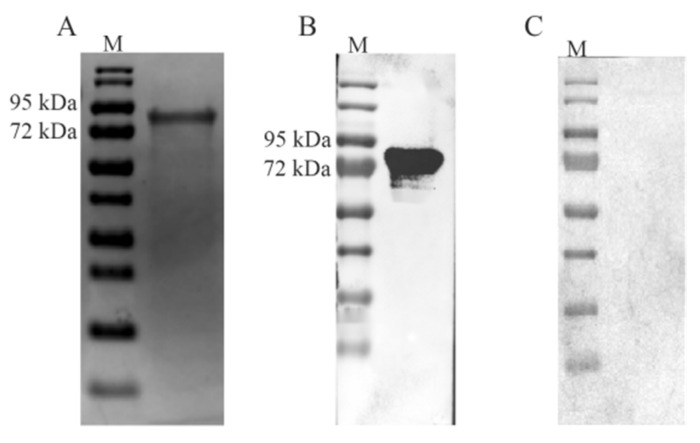
Purification and reactogenicity of *Et*G6PI. (**A**) SDS-PAGE analysis of purified r*Et*G6PI; (**B**) r*Et*G6PI was probed with the rabbit anti-sporozoites serum; (**C**) r*Et*G6PI was probed with the negative rabbit serum.

A recombinant expression plasmid pGEX-4T-*Et*G6PI was constructed and induced to express the recombinant protein in *E. coli*. SDS-PAGE confirmed that the recombinant protein (r*Et*G6PI) was soluble. After purification by affinity chromatography, a protein band of approximately 74.5 kDa was observed with SDS-PAGE (Figure 3A). Western blotting detected specific bands at the expected size when probed with rabbit anti-sporozoite serum (Figure 3B). No bands were detected when using rabbit healthy serum as the primary antibody (Figure 3C). These results indicate that r*Et*G6PI exhibits good immunoreactivity.

### 3.3. Transcription and Translation of EtG6PI at E. tenella Different Developmental Stages

**Figure 4:**
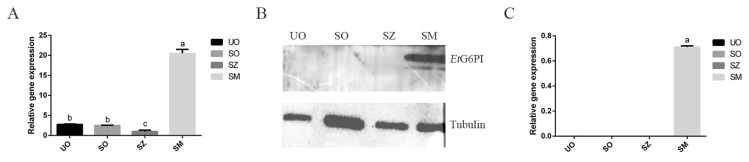
mRNA transcription and translation of *Et*G6PI in different development stages of *E. tenella*. (**A**) mRNA transcriptional levels; (**B**) translation level; (**C**) grayscale analysis. UO, unsporulated oocyst; SO, sporulated oocyst; SZ, sporozoite; SM, second-generation merozoite. (**A**,**C**) Different lowercase letters (a, b, c) indicate significant differences among groups (*p* < 0.05, Tukey’s multiple comparisons test). Data are presented as mean ± SD; *n* = 3 biological replicates (with three technical replicates).

The mRNA transcription levels of *Et*G6PI in four developmental stages of the *E. tenella* DS strain was detected by qPCR using 18S rRNA as the internal reference gene. The results showed that the mRNA transcription level of *Et*G6PI was highest in second-generation merozoites, while it was lower in the other three stages (unsporulated oocysts, sporulated oocysts and sporozoites) (Figure 4A).

Using mouse anti-α-Tubulin as control, the rabbit anti-r*Et*G6PI polyclonal serum was used as the primary antibody to incubate with the extracted parasite proteins from the four stages of the DS strain for Western blotting. The results showed that the protein translation level of *Et*G6PI was the highest in second-generation merozoites and lowest in the other three stages, which was consistent with the qPCR results (Figure 4B,C).

### 3.4. Transcription of EtG6PI in Maduramycin-Resistant Strains and Drug-Sensitive Strain of E. tenella

**Figure 5:**
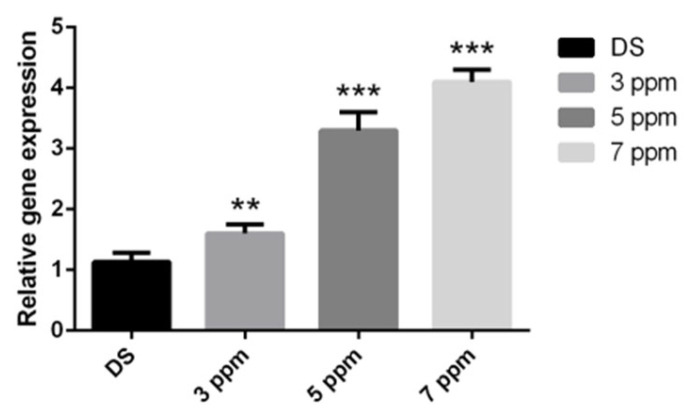
mRNA transcription levels of *Et*G6PI at different concentrations of maduramycin-resistant strains. DS, drug-sensitive strain; 3 ppm, 3 ppm maduramycin-resistant strain; 5 ppm, 5 ppm maduramycin-resistant strain; 7 ppm, 7 ppm maduramycin-resistant strain; ppm, mg/kg. **, very signifificant differences (0.001 < *p* < 0.01); ***, highly significant differences (*p* < 0.001). Data are presented as mean ± SD; *n* = 3 biological replicates (with three technical replicates). *p* (DS vs. 3 ppm) = 0.0195, *p* (DS vs. 5 ppm) = 0.0004, *p* (DS vs. 7 ppm) < 0.0001.

Using cDNA from sporozoites and 18S rRNA as a reference, we analyzed the mRNA transcription level of *Et*G6PI by qPCR across the DS strain and maduramycin-resistant strains with varying resistance levels. The results showed that *Et*G6PI transcription was not only significantly upregulated in the MRR strain compared to the DS strain, but it also exhibited an increasing trend correlating with the degree of drug resistance. *Et*G6PI expression was ~3.6-fold higher in the 7 ppm MRR strain than in DS (*p* < 0.001) (Figure 5).

### 3.5. Subcellular Localization of EtG6PI in E. tenella

**Figure 6:**
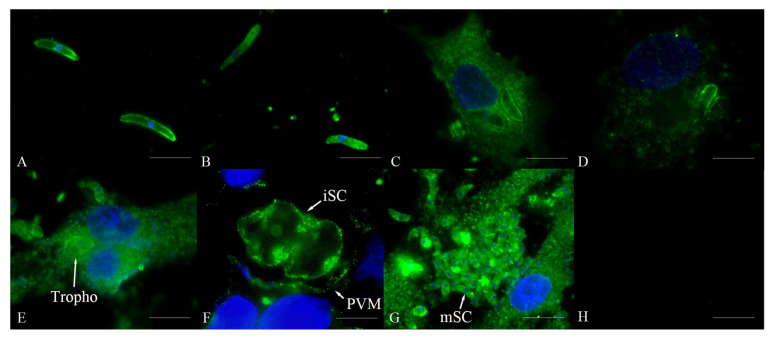
Location of *Et*G6PI in different developmental stages of *E. tenella*. (**A**) sporozoites in PBS; (**B**) second-generation merozoite in PBS; (**C**) 2 h post-infection; (**D**) 12 h post-infection; (**E**) 24 h post-infection; (**F**) 60 h post-infection; (**G**) 72 h post-infection; (**H**) negative control, healthy rabbit IgG as the primary antibody; bar = 10 μm; Tropho, trophozoites; iSC, immature schizonts; PVM, parasitophorous vacuole membrane; mSC, mature schizonts. Consistent exposure conditions were applied across all images.

The subcellular localization of *Et*G6PI was investigated in free sporozoites, second-generation merozoites, and during intracellular development in DF-1 cells using indirect immunofluorescence assay. In sporozoites and merozoites, *Et*G6PI was localized to the surface and cytoplasm, with particularly intense fluorescence at the anterior end of sporozoites (Figure 6A,B).

At 2 h post-invasion, the protein remained predominantly on the surface, apex, and within the cytoplasm of the invading parasites (Figure 6C). As the infection progressed, the apical fluorescence intensity diminished, and the protein localized mainly to the parasite surface and cytoplasm (Figure 6D,E). By 60 h post-invasion *Et*G6PI was detected not only on the parasite (immature schizont) surface and cytoplasm but also on the PVM (Figure 6F). As the parasites developed into first-generation merozoites, the fluorescence intensity increased again, and the protein was localized on the surface and in the cytoplasm of these merozoites (Figure 6G). No specific fluorescence was observed in the negative control group (Figure 6H).

### 3.6. Anti-rEtG6PI Antibody Inhibits E. tenella Sporozoite Invasion In Vitro in a Dose-Dependent Manner

**Figure 7:**
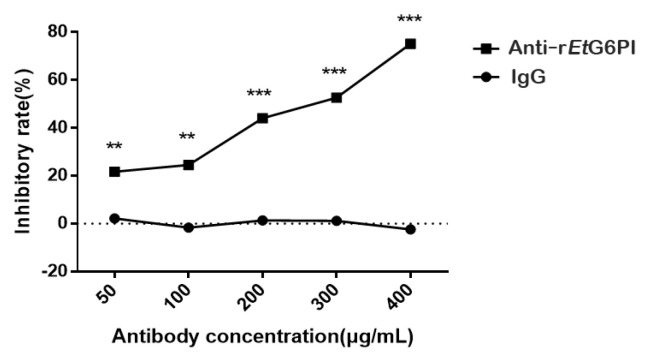
Effect of sporozoites invasion in vitro by rabbit anti-r*Et*G6PI. Dotted line: 0% inhibitory rate; **, very signifificant differences (0.001 < *p* < 0.01); ***, highly significant differences (*p* < 0.001) (one-way ANOVA with Tukey’s post hoc test). *n* = 3 biological replicates (with three technical replicates).

To assess the effect of anti-r*Et*G6PI antibody on sporozoite invasion, sporozoites were pre-incubated with IgG at various concentrations prior to inoculation into DF-1 cells. IgG from healthy rabbits served as a negative control. The healthy rabbit IgG showed no significant inhibitory effect on invasion. In contrast, the anti-r*Et*G6PI antibody inhibited invasion in a dose-dependent manner. As the antibody concentration increased from 50 to 400 µg/mL, the inhibition rate rose progressively from 21% to approximately 75% (Figure 7).

### 3.7. Detection Transcription Level of EtG6PI in Field Samples

**Figure 8:**
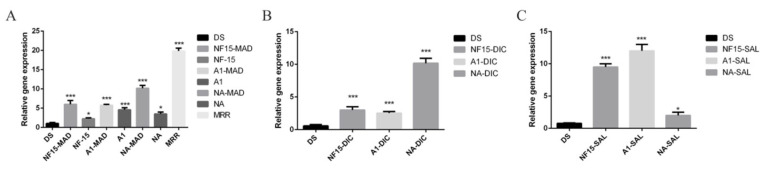
mRNA transcriptional level analysis of *Et*G6PI in different field isolates and resistant strains. (**A**) field isolates and maduramycin-resistant isolates; (**B**) diclazuril-resistant isolates; (**C**) salinomycin-resistant isolates. DS, drug-sensitive strain; MRR, maduramycin-resistant strain; NF15, Nantong F15 field isolates; A1, Anhui 1 field isolates; NA, Nantong A field isolates; MAD, maduramycin-treated; DIC, diclazuril-treated; SAL, salinomycin-treated. *: *p* < 0.05; ***: *p* < 0.001. Data are presented as mean ± SD; *n* = 3 biological replicates (with three technical replicates). *p* values vs. DS are shown: (**A**) NF15-MAD (*p* = 0.0002), NF-15 (*p* = 0.019), A1-MAD (*p* < 0.0001), A1 (*p* = 0.0005), NA-MAD (*p* < 0.0001), NA (*p* = 0.012), MRR (*p* < 0.0001); (**B**) NF15-DIC (*p* = 0.0001), A1-DIC (*p* = 0.0005), NA-DIC (*p* < 0.0001); (**C**) NF15-SAL (*p* < 0.0001), A1-SAL (*p* < 0.0001), NA-SAL (*p* = 0.0141).

To establish a new method for detecting drug resistance in field of *Eimeria* spp., three field isolate strains (NF15, A1, NA) were investigated. All three field isolates exhibited complete resistance to diclazuril, maduramycin and salinomycin. The mRNA transcription levels of *Et*G6PI were compared among the DS strain, mixed field strains, diclazuril-resistant field isolates, maduramycin-resistant field isolates, and salinomycin-resistant field isolates. The results indicated that the mRNA transcription levels of *Et*G6PI in the three mixed field isolates, three diclazuril-resistant field isolates, three maduramycin-resistant field isolates and three salinomycin-resistant field isolates were significantly higher than those in the DS strain (e.g., A1 vs. DS: ~4.8-fold, NA-MAD vs. DS: ~9.5-fold, NA-DIC vs. DS: ~19-fold, A1-SAL vs. DS: 14.6-fold) (Figure 8).

## 4. Discussion

In this study, we amplified and sequenced the *Et*G6PI ORF. BLASTp analysis showed that its encoded protein shares 96% amino acid identity with the glucose-6-phosphate isomerase from *E. necatrix*. Phylogenetic analysis further confirmed their close evolutionary relationship. This high sequence conservation likely reflects both the close genetic relationship between *E. tenella* and *E. necatrix* and the functional importance of the enzyme [20]. Notably, both *Eimeria* species parasitize the cecal epithelium of chickens, suggesting that adaptation to this common host microenvironment may have contributed to the conservation of this key metabolic gene.

IFA revealed that *Et*G6PI localizes to the parasite surface, cytoplasm, and PVM. Its presence on the PVM suggests non-canonical secretion, despite the absence of a signal peptide or transmembrane domain. Given that the PVM, a critical host–parasite interface formed during invasion, protects the parasite and mediates nutrient exchange [21], we hypothesize that *Et*G6PI may participate in host–parasite interactions, potentially aiding invasion or immune evasion. G6PI is a well-established moonlighting protein with diverse functions beyond glycolysis. In mammals, for example, it can be secreted and act as a trophic factor. This is supported by studies in a mouse model of fatty liver disease, where secreted G6PI detected in serum was shown to bind the AMFR/gp78 receptor [22]. Consistent with its extracellular role, inhibition of G6PI also reduces the proliferation, migration, and invasion of fibroblast-like synoviocytes [12]. The functional versatility of G6PI is conserved across kingdoms. In the pathogenic bacterium *Acidovorax citrulli*, it contributes to virulence, biofilm formation, motility, and stress tolerance [23]. Similarly, in the fungus *Cryptococcus neoformans*, G6PI is essential for virulence-associated traits such as melanin and capsule biosynthesis, cell wall integrity, and stress resistance [24]. Supporting this functional versatility, our in vitro invasion inhibition assay demonstrated that anti-r*Et*G6PI antibody significantly impedes the invasion of *E. tenella* sporozoites. We therefore postulate that the polyclonal antibodies bind to the *Et*G6PI protein localized on the parasite surface, disrupting its protective function and consequently inhibiting host cell invasion. Nevertheless, due to the unavailability of monoclonal antibodies at present, the use of polyclonal antibodies without pre-absorption controls limits the certainty of target specificity. Future studies using monoclonal antibodies or pre-absorbed sera are needed to confirm that the observed invasion inhibition is solely attributable to *Et*G6PI neutralization. Given the sequence similarity between *Et*G6PI and host G6PI, anti-*Et*G6PI antibodies may cross-react with host G6PI in vivo. This is a potential concern for any therapeutic application and should be evaluated in future studies using host cell-based assays or in vivo models.

We analyzed the transcription and translation levels of *Et*G6PI across *E. tenella* developmental stages using qPCR and Western blotting. The results showed that *Et*G6PI was highly expressed in second-generation merozoites, while its expression was relatively low in unsporulated oocysts, sporulated oocysts, and sporozoites. Indirect immunofluorescence further revealed that the fluorescence signal of *Et*G6PI intensified during schizogony (from immature schizonts to first-generation merozoites) within DF-1 cells, indicating elevated expression at these stages. The schizogony of *Eimeria* occurs in a hypoxic (anaerobic) host environment and has high energy (ATP) demands. Glycolysis likely serves as a primary or sole energy source under these conditions, which would necessitate the upregulation of key enzymes such as *Et*G6PI. This hypothesis is consistent with observations in other biological systems. For example, in the hypoxic synovial tissue of rheumatoid arthritis (RA) patients, G6PI expression is elevated, and hypoxia can directly induce G6PI expression and proliferation in synovial fibroblasts [25]. Similarly, in glioblastoma, silencing phosphoglucose isomerase/autocrine motility factor (PGI/AMF) inhibits tumorigenesis, highlighting its role in regulating cancer stem cell proliferation [26]. So, we speculate that the upregulation of *Et*G6PI is associated with the development and proliferation of *E. tenella* during its in vivo stages, serving as an adaptation to the hypoxic and energy-intensive conditions of schizogony.

In addition, based on the finding that *Et*G6PI mRNA is significantly upregulated in the maduramycin-resistant strain, we hypothesize that this upregulation contributes to drug resistance. Maduramycin, an ionophore, kills parasites by disrupting ion gradients across the cell membrane by binding monovalent ions (e.g., K^+^, Na^+^) [27]. To counteract this, the parasite must activate ATP-dependent ion pumps (e.g., Ca^2+^/Mg^2+^-ATPase, Na^+^/K^+^-ATPase) to restore homeostasis, a process demanding substantial energy [28]. The upregulation of *Et*G6PI is therefore posited to enhance ATP production to meet this heightened energy demand. This hypothesis is supported by analogous roles of G6PI in other systems. In enzalutamide-resistant prostate cancer, G6PI overexpression mediates therapeutic resistance [29]. Conversely, in *Vibrio cholerae*, deletion of the G6PI gene increases susceptibility to β-lactam antibiotics [30]. G6PI’s role extends beyond glycolysis to maintaining cellular integrity in bacteria and fungi [31], suggesting dual potential benefits for a resistant parasite. Supporting the relevance of glycolysis in this resistance, overexpression of another glycolytic enzyme, phosphofructokinase (*Et*PFK1), has also been shown to confer maduramycin resistance in *E. tenella* [32]. So, we propose two non-mutually exclusive mechanisms by which elevated *Et*G6PI expression could hypothetically confer resistance. First, by enhancing cellular membrane integrity to reduce drug-induced damage; and second, by increasing glycolytic flux and ATP production to fuel active drug efflux or detoxification systems, thereby accelerating drug elimination. While these hypotheses provide a framework, the precise role of *Et*G6PI in *E. tenella* drug resistance remains to be fully elucidated. Direct measurements of intracellular ATP levels, membrane potential, and drug accumulation in DS and MRR strains, with or without *Et*G6PI modulation, will be required to test these models in future studies. The recent development of CRISPR-Cas9 gene editing for *Eimeria* spp. now provides a powerful tool to directly test these models and uncover the molecular basis of resistance in the future. In the present study, we provide correlative evidence linking *Et*G6PI upregulation to maduramycin resistance. Definitive functional validation (e.g., CRISPR-Cas9 knockout or overexpression) will be required to establish causality, which is underway in our laboratory.

The control of avian coccidiosis through chemotherapy is fundamentally challenged by widespread drug resistance. Current diagnosis relies solely on cumbersome in vivo drug sensitivity tests, which are expensive, time-consuming, and often only confirm resistance after it has already compromised flock health, thereby hindering timely intervention [33]. This diagnostic delay highlights the urgent need for molecular markers capable of preemptive resistance detection. Our study identified *Et*G6PI as differentially upregulated in laboratory-induced maduramycin-resistant strains, with expression levels increasing alongside drug concentration. To evaluate its field relevance, we analyzed three field isolates (NF15, A1, NA) with documented multi-drug resistance [19]. All field strains showed significantly elevated *Et*G6PI expression compared to DS strain. This upregulation was observed not only under maduramycin pressure but also in association with resistance to other anticoccidials, including salinomycin and diclazuril. Interestingly, transcriptional analysis revealed no significant upregulation of *Et*G6PI in laboratory-generated diclazuril-resistant (DZR) or salinomycin-resistant (SMR) sporozoites compared to DS [34]. This contrast suggests that *Et*G6PI upregulation in field isolates likely results from complex, multi-drug resistance profiles rather than being a specific response to any single compound. This is likely due to the distinct selection pressures between field and laboratory environments. Field isolates were exposed to complex drug-use histories (e.g., rotation and combination), which confer a survival advantage to parasites with cross-resistance mechanisms [35]. The elevated *Et*G6PI expression in field isolates may reflect maduramycin resistance within their multidrug profiles, associate with resistance to other drugs (e.g., diclazuril and salinomycin), or represent a broader metabolic adaptation to complex drug pressures. Therefore, while *Et*G6PI shows promise as a biomarker for identifying maduramycin resistance in field strains, it cannot reliably predict resistance to other drugs alone. Future studies should combine other markers for joint detection and use laboratory-generated strains resistant to individual drugs to distinguish these possibilities.

In summary, this study provides novel evidence linking *Et*G6PI to both host cell invasion and maduramycin resistance in *E. tenella*. However, several limitations should be noted: the lack of direct functional validation (e.g., gene manipulation), the correlational nature of resistance data, and the undefined specificity of the antibody used. Our results reveal stage-specific expression of *Et*G6PI, with both transcriptional and protein levels peaking in second-generation merozoites. *Et*G6PI transcription is upregulated in maduramycin-resistant strains under both laboratory and field conditions, supporting its potential as a biomarker for field detection of maduramycin resistance. Indirect immunofluorescence localization showed that *Et*G6PI localizes to the parasite surface, cytoplasm, and PVM, with fluorescence intensity increasing during intracellular development. In addition, in vitro inhibition assays confirmed that anti-r*Et*G6PI antibodies significantly impair sporozoite invasion of host cells. Together, these findings suggest *Et*G6PI as a candidate for further functional validation for elucidating the metabolic mechanisms of coccidian drug resistance and for developing novel molecular detection tools.

## Data Availability

The original contributions presented in this study are included in the article. Further inquiries can be directed to the corresponding authors.

## Abbreviations

The following *E. tenella* strains abbreviations are used in this manuscript:

DS	drug-sensitive strain
MRR	maduramycin-resistant strain
DZR	diclazuril-resistant strain
SMR	salinomycin-resistant strain
NF15	Nantong F15 field isolates
A1	Anhui 1 field isolates
NA	Nantong A field isolates
NF15-MAD	Nantong F15 field isolates treated with maduramycin
A1-MAD	Anhui 1 field isolates treated with maduramycin
NA-MAD	Nantong A field isolates treated with maduramycin
NF15-DIC	Nantong F15 field isolates treated with diclazuril
A1-DIC	Anhui 1 field isolates treated with diclazuril
NA-DIC	Nantong A field isolates treated with diclazuril
NF15-SAL	Nantong F15 field isolates treated with salinomycin
A1-SAL	Anhui 1 field isolates treated with salinomycin
NA-SAL	Nantong A field isolates treated with salinomycin
